# Spin-momentum locked spin manipulation in a two-dimensional Rashba system

**DOI:** 10.1038/s41598-018-37967-9

**Published:** 2019-02-13

**Authors:** Makoto Kohda, Takanori Okayasu, Junsaku Nitta

**Affiliations:** 1 Department of Materials Science, Tohoku University, 6-6-02 Aramaki-Aza Aoba, Aoba-ku Sendai, 980-8579 Japan; 2 Center for Spintronics Research Network, Tohoku University, 2-1-1 Katahira, Aoba-ku Sendai, 980-8577 Japan; 3 Center for Science and Innovation in Spintronics (Core Research Cluster), Tohoku University, 2-1-1 Katahira, Aoba-ku Sendai, 980-8577 Japan

## Abstract

Spin-momentum locking, which constrains spin orientation perpendicular to electron momentum, is attracting considerable interest for exploring various spin functionalities in semiconductors and topological materials. Efficient spin generation and spin detection have been demonstrated using the induced helical spin texture. Nevertheless, spin manipulation by spin-momentum locking remains a missing piece because, once bias voltage is applied to induce the current flow, the spin orientation must be locked by the electron momentum direction, thereby rendering spin phase control difficult. Herein, we demonstrate the spin-momentum locking-induced spin manipulation for ballistic electrons in a strong Rashba two-dimensional system. Electron spin rotates in a circular orbital motion for ballistically moving electrons, although spin orientation is locked towards the spin-orbit field because of the helical spin texture. This fact demonstrates spin manipulation by control of the electron orbital motion and reveals potential effects of the orbital degree of freedom on the spin phase for future spintronic and topological devices and for the processing of quantum information.

## Introduction

**Figure 1:**
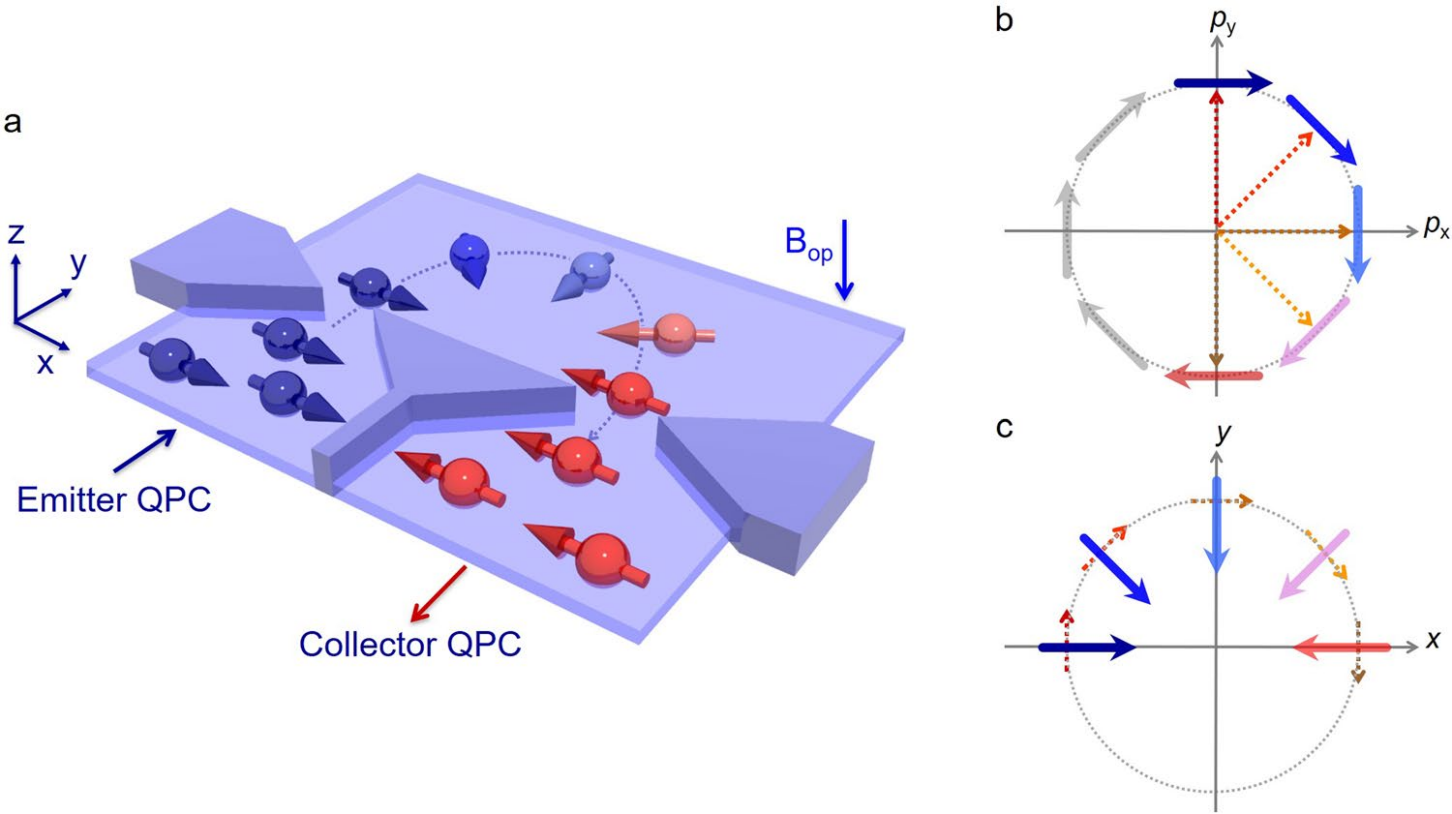
Spin-momentum locking-induced spin manipulation under transverse magnetic focusing. (**a**) The InGaAs-based two-dimensional electron gas is processed to lateral quantum point contacts (QPCs). Electron spins are polarized and detected through these QPCs because of the spatial gradient of the spin-orbit (SO) field. Under the weak out-of-plane magnetic field *B*_op_, generated spin-polarized electrons from the emitter QPC are focused to the collector QPC by the Lorentz force. During the orbital trajectory, the SO field constrains the electron spin orientation perpendicular to the momentum direction by spin-momentum locking. The spin orientation takes the opposite direction to that of the spins generated in the emitter QPC. (**b**) Spin orientations in momentum space based on equation (1). When the momentum changes from +*p*_y_ to −*p*_y_ direction with clockwise rotation, spin orientation also rotates from right to left. (**c**) Orbital motion (dotted arrows) as well as spin orientation (solid arrows) in real space described in (**b**).

The coupling of electron spin and orbital motion provides various spin functionalities: from spin-charge conversion to magnetization reversal^1–6^. Rashba spin-orbit (SO) interaction^7^ plays a central role in this coupling to lock the spin orientation perpendicular to the electron momentum^8^. Topological insulators also have such a relation between electron’s spin and momentum hosted by a spin-polarized massless Dirac state^3,9–12^. This coupling is known as ‘spin-momentum locking’. Because the spin orientation is forced to align perpendicularly to the electron momentum, *i.e*., the current direction, remarkable efforts have been undertaken for efficient spin generation and/or spin detection by controlling the flow of charge or spin currents^13–16^. Such generation and detection constitute important building blocks for future spintronics and topological electronics. Moreover, spin manipulation becomes indispensable for a full set of spin controls based on spin-momentum locking. However, because the spin orientation is constrained by the electron momentum direction, changing the spin orientation under a biased current, such as those of drift and diffusion electron motions, makes it difficult. Therefore, spin manipulation using spin-momentum locking has been eagerly pursued. In a ballistic regime, where the electron trajectory plays a fundamentally important role, spin orientation can be rotated by modulating electron orbital motion because spin follows along the SO field direction under the ballistic orbital trajectory^17,18^. To realize such spin manipulation in a ballistic regime, the long mean free path and strong SO interaction are required. Although both graphene and GaAs two-dimensional (2D) electron gas have a sufficiently long mean free path, spin manipulation by spin-momentum locking is difficult to observe in their weak SO fields^19,20^. Although some topological materials show strong SO interaction and a long mean free path on the surface state^21,22^, separation of bulk and surface conduction remains challenging in epitaxial growth without compromising mobility. GaAs 2D hole gas is suitable because of its long mean free path and strong SO interaction^23–25^. The lateral magnetic focusing has revealed the spin separation from unpolarized electron current since the SO interaction induces different Fermi wave vectors for spin-up and spin-down electrons, making the different cyclotron trajectories^23^, and also enabled to evaluate the spin polarization in a 0.7 conductance anomaly by using the spin separation technique^24,25^. However, spin manipulation by spin-momentum locking has yet to be demonstrated. In the present study, we experimentally manifested spin manipulation by spin-momentum locking in a magnetic focusing device. In a 2D system with strong SO interaction, spin orientation is directed preferentially to the SO-induced magnetic field. It remains focused in this direction due to spin-momentum locking. Because the Rashba SO field depends on the direction of electron momentum, spin orientation is manipulated by modulating the electron orbital motion (Fig. 1a). This result demonstrates spin manipulation by spin-momentum locking in the ballistic regime. Because spin-momentum locking exists ubiquitously not only in semiconductors but also in topological insulators, oxides, and metal interfaces, it provides a new direction for exploring spin manipulation, especially in strong SO materials.

In the III–V semiconductor quantum well (QW), the Rashba SO Hamiltonian is described as^7^

1 $${H}_{R}=\frac{\alpha }{\hslash }({p}_{y}{\sigma }_{x}-{p}_{x}{\sigma }_{y}),$$

where *α* is the Rashba SO coefficient proportional to the strength of the internal electric field along the growth direction of a QW (*α* > 0 for present QW), *σ*_*i*_ (*i* = *x*, *y*) are the *x* and *y* components of the Pauli spin matrix with *x*//[110] and *y*//[1–10], and the in-plane momentums *p*_x_ and *p*_y_ are defined respectively as ${p}_{x}=p\,\cos \,\theta$ and ${p}_{y}=p\,\sin \,\theta$, where *p* represents the electron momentum and where *θ* is counted from the *x*-axis. The corresponding spin orientation becomes perpendicular to the direction of electron momentum because of spin-momentum locking. It exhibits a helical spin texture (Fig. 1b). To manipulate spin orientation by spin-momentum locking, let us consider the rotational change in the electron momentum from +${\overrightarrow{p}}_{y}$ to −${\overrightarrow{p}}_{y}$ in a clockwise rotation (dotted arrows in Fig. 1b). The spin orientation, initially to the right in +${\overrightarrow{p}}_{y}$, rotates clockwise as the *p* direction changes. It is eventually directed to the left in −${\overrightarrow{p}}_{y}$. This momentum modulation corresponds to a semicircular trajectory in real space (Fig. 1c), where the spin orientation switches to the opposite direction after orbital motion. Because the frequency of spin precession under the SO field is faster than the cyclotron frequency, the adiabatic limit of spin rotation is satisfied, making it possible to lock the spin orientation in the direction of the SO field^17,18^.

We detect this spin-momentum locked spin manipulation by using spin-polarized transverse magnetic focusing in an InGaAs/InGaAsP semiconductor 2D electron gas. As presented in Fig. 1a, the Lorentz force of a weak out-of-plane external magnetic field *B*_op_ to the QW plane determines the path of ballistic electrons from the emitter to collector constrictions. From emitter to collector, the focusing time of these electrons travelling at Fermi velocity is only a few picoseconds, minimizing the influence of spin relaxation^26^. Two narrow constrictions, the so-called lateral quantum point contacts (QPCs), enable us to polarize and detect the in-plane spin component because of the strong SO interaction^27^. We first confirmed the spin polarization and detection functionalities in a single QPC by elucidating the subband energy. We then investigated the stability of spin polarization under *B*_op_ fields of various strengths. Finally, we explored the spin orientation using transverse magnetic focusing in a lateral QPC device. Comparison of this focusing signal with that from a Monte Carlo simulation revealed spin manipulation by spin-momentum locking.

## Results

### Spin polarization/detection functionalities and stability of spin polarization under perpendicular external magnetic fields in a single QPC

**Figure 2:**
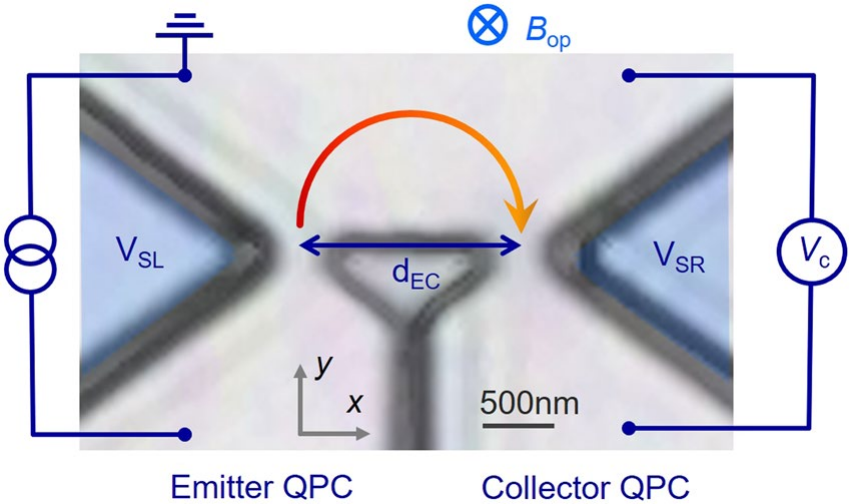
Measurement setup for lateral QPC device. Two trench-type QPCs are connected in parallel as the emitter (left) and collector (right). Side gate bias voltages *V*_SR_ and *V*_SL_ control the channel conductance. The entire structure is covered with an Al_2_O_3_ insulator/Cr/Au as the top gate to control the mean free path and Rashba SO field. Electrons from the emitter QPC are focused to the collector QPC by the out-of-plane magnetic field *B*_op_, resulting in the accumulation of electrons in the collector QPC. This accumulation can be measured as the collector voltage *V*_c_ across the collector QPC. Distance between emitter and collector QPCs is defined as *d*_EC_.

Figure 2 portrays a fabricated lateral QPC device for magnetic focusing with measurement configurations (also see Device Structure and Fabrication in Methods). An InGaAs/InGaAsP 10 nm QW was used to realize a large Rashba SO field on the order of 12 T^28^ to satisfy the adiabatic limit of spin rotation. An epitaxial wafer was processed to produce trench-type single and lateral QPCs. The top gate electrode was deposited to obtain a long mean free path and to modulate the Rashba SO coefficient *α*^29^. The distance between the emitter and collector in the lateral QPC was designed as *d*_EC_ = 1.4 μm. Quantized conductance was measured by sweeping the side-gate bias voltage (*V*_SR_ and *V*_SL_) under constant top gate *V*_TG_. We subtract the series resistance (680–1200 Ω) in the QPC by evaluating the sheet resistance in the Hall bar and taking into account the side wall scattering by the QPC geometry. For transverse magnetic focusing, the voltage *V*_c_ across the collector QPC was measured under a fixed emitter current *I*_*e*_ = 100 nA. The temperature was *T* = 0.22–1.5 K.

**Figure 3:**
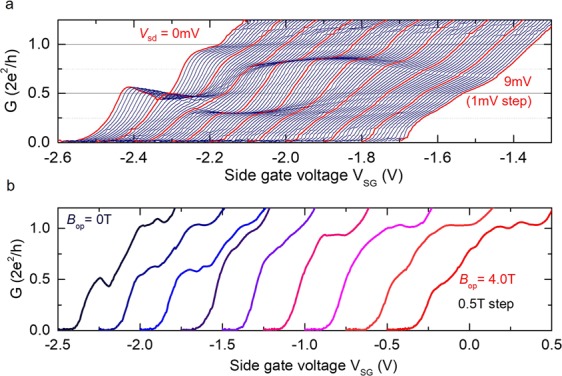
Quantized conductance and out-of-plane magnetic field dependence in a single QPC. (**a**) Conductance *G* (in unit of 2*e*^2^*/h*) as a function of side gate bias voltage *V*_SG_ with different dc source–drain bias voltage *V*_sd_ (0.1 mV step from 0 mV to 9 mV) at *B*_op_ = 0 T and *T* = 0.22 K. The top gate bias voltage is fixed at *V*_TG_ = +0.3 V; the corresponding Rashba SO coefficient *α* is 3.74 × 10^−12^ eVm. Thick red lines correspond to each 1 mV step from 0 mV (far left) to 9 mV (far right). (**b**) Quantized conductance with different out-of-plane *B*_op_ field from 0 to 4.5 T with 0.5 T step. *T* = 0.22 K and *V*_TG_ = +0.3 V.

Spontaneous spin polarization in a side-gated QPC is a prerequisite for both the alignment and detection of spin orientation in magnetic focusing. Therefore, we first investigated the spin polarization and detection functionalities in a single QPC by clarifying spin splitting. The subband energy in QPCs can be investigated by application of a dc source–drain bias voltage (*V*_sd_) because the energy separation of *eV*_sd_ provides a difference in the chemical potentials of electrons travelling to the left and right^30,31^. Figure 3a shows quantized conductance *G* (in units of 2*e*^2^*/h*) as a function of the side gate voltage *V*_SG_ with different *V*_*sd*_ at *B*_op_ = 0 T and *T* = 0.22 K. At *V*_sd_ = 0 mV (the red line at the far left in Fig. 3a), a 0.5(2*e*^2^*/h*) plateau is apparent in addition to plateaus at integer (*N*) multiples of 2*e*^2^*/h* (*N* ≥ 2 is not shown): this plateau is a clear signature of the spin resolved state. The conductance oscillation around 0.5(2*e*^2^*/h*) plateau is owing to the interference effect of the coherent electron^32^. As *V*_sd_ increases from 0 to 9 mV, the 0.5(2*e*^2^*/h*) plateau gradually weakens and disappears at *V*_sd_ = 2 mV. Instead, plateaus around 0.25(2*e*^2^*/h*) and 0.75(2*e*^2^*/h*) develop for 2 mV ≤ *V*_sd_ ≤ 5 mV. Then the 0.5(2*e*^2^*/h*) plateau reappeared with the further application of *V*_sd_ = 9 mV. This plateau evolution on *V*_sd_ can be understood by considering the spin-split subband formation in a zero magnetic field. For small e*V*_sd_, conductance shows half-integer plateaus as $$1/2\times N(2{e}^{2}/h)$$ because of the identical numbers of the spin-split subband below the source and drain chemical potentials. With increasing *eV*_sd_, the subband numbers differ by one. Then, the average conductance for the source and drain yields quarter-integer plateaus in accordance with $$0.5\,[1/2(N+1)(2{e}^{2}/h)+1/2\,N(2{e}^{2}/h)]=$$$$(1/2N+1/4)(2{e}^{2}/h)$$. Such quarter-integer plateaus have evolved in GaAs/AlGaAs QWs by application of a large in-plane magnetic field *B*_in_^30,31,33^. In the present device, the plateaus at 0.25(2*e*^2^*/h*) and 0.75(2*e*^2^*/h*) without *B*_in_ indicate an appreciable spin gap energy. According to this scenario, when the subband numbers differ by two, then $$0.5\,[1/2(N+2)(2{e}^{2}/h)+1/2N(2{e}^{2}/h)]=1/2(N+1)(2{e}^{2}/h)$$; the half-integer plateau reappears in larger e*V*_sd_. This reappearance, which corresponds to the case of *V*_sd_ = 9 mV, is consistent with the constant spin energy gap. As a result, spontaneous spin polarization is formed in the QPC device. We have estimated the spin-split energy gap as 5–6 meV at *T* = 0.22 K from source-drain bias dependence of differential conductance^27^. The spin polarization mechanism in such a strongly SO-coupled QPC was confirmed in an earlier study^27^. The spatial gradient of the SO field generates a spin-dependent force, spatially separating spin up and spin down electrons. Because the Rashba SO field is in an in-plane direction and perpendicular to electron momentum, in-plane and collinear spin polarization to the SO field is generated through the constriction.

The stability of the 0.5(2*e*^2^*/h*) plateaus under the *B*_op_ field was checked to ensure that spin polarization was maintained during magnetic focusing. Figure 3b shows quantized conductance with different *B*_op_ fields at *T* = 0.22 K. Both 0.5(2*e*^2^*/h*) and 1.0(2*e*^2^*/h*) plateaus are observed stably up to 1.0 T, indicating that induced orbital modulation in such a small *B*_op_ field does not affect the stability of the spin polarized plateau. However, at 1.5 T ≤ *B*_op_ ≤ 2.0 T, both plateaus disappear, which is likely because of the mixing of the spin channels by orbital modulation. The 0.5(2*e*^2^*/h*) plateau is, on the other hand, stably remained up to ±8 T under the in-plane external magnetic field (Detailed results are in Supplementary Information). Then with the further application of *B*_op_, well developed 1.0(2*e*^2^*/h*) and small 0.5(2*e*^2^*/h*) plateaus appear because of Zeeman splitting and additional confinement by cyclotron motion. Because the main focusing peak for spin manipulation by spin-momentum locking appears at around *B*_op_ = 0.3 T, which is much smaller than 1.5 T at which the 0.5(2*e*^2^*/h*) plateau disappears, we can safely exclude spin depolarization by *B*_op_.

### Spin-momentum locked spin manipulation revealed by transverse magnetic focusing in a lateral QPC device

**Figure 4:**
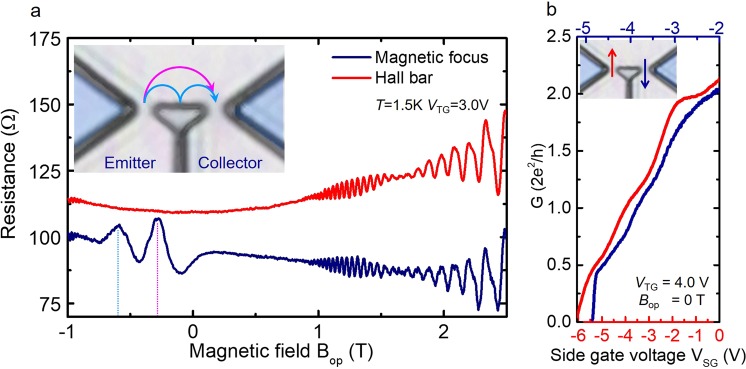
Transverse magnetic focusing without spin polarization and quantized conductance in a lateral QPC device. (**a**) Transverse magnetic focusing measured without spin resolution (*V*_SR_ = *V*_SL_ = 0 V) at *V*_TG_ = 3.0 V (blue line). The measurement temperature was 1.5 K. Resistance of collector constriction is detected while sweeping *B*_op_ field between −1 to +2.5 T with constant bias current *I*_e_ = 100 nA applied in emitter constriction. The red line corresponds to longitudinal resistance measured simultaneously in Hall bar connected to the lateral QPC. (**b**) Conductance of emitter (red line) and collector (blue line) QPCs as a function of *V*_SR_ and *V*_SL_ at *T* = 1.5 K. The top gate is fixed at *V*_TG_ = 4.0 V, where *α* = 6.0 × 10^−12^ eVm. Both QPCs show the spin polarized plateau at around 0.5(2*e*^2^*/h*).

Based on the spin properties investigated in the single QPC, we designed the lateral QPC device for transverse magnetic focusing presented in Fig. 2. Because ballistic orbital motion is fundamentally important to lock the spin orientation, we first verify the orbital trajectory for electrons without spin resolution (*V*_SR_ = *V*_SL_ = 0 V) at *V*_TG_ = +3.0 V by sweeping the *B*_op_ field. The measured resistance across the collector constriction is depicted in blue in Fig. 4a. We also took simultaneous measurements of the longitudinal resistance in the Hall bar connected to the lateral QPC (shown as red in Fig. 4a) and found Shubnikov de-Haas (SdH) oscillation for both the lateral QPC and Hall bar. Spin splitting caused by the SO interaction was detected by the beating pattern, which started at around *B*_op_ = +1.0 T. In the negative *B*_op_ field, where the electron is travelling towards the collector constriction, collector resistance shows peaks at *B*_op_ = −0.28 and −0.59 T before SdH oscillation starts. The first peak at *B*_op_ = −0.28 T corresponds to the direct incoming electrons from the emitter constriction, satisfying the condition for matching the cyclotron diameter 2*r*_c_ to the emitter–collector distance *d*_EC_. The second peak at *B*_op_ = −0.59 T originates from the electrons reflected at the sample edge, which then refocus the collector. The values of *r*_c_ evaluated from the first and second peaks are, respectively, 0.77 and 0.76 μm, which correspond to the designed emitter–collector distance (*d*_EC_/2 = 0.7 μm).

To functionalize the spin properties, we applied the side gate voltage to induce quantized conductance in the lateral QPCs. Figure 4b presents the conductance of the emitter (as red) and the collector (as blue) QPCs as a function of right and left side gate *V*_SR/SL_, respectively, at *T* = 1.5 K. The top gate was fixed at *V*_TG_ = 4.0 V (*α* = 6.0 × 10^−12^ eVm). The quantized conductance with a 0.5(2*e*^2^*/h*) plateau reproduced in both QPCs confirms the spin-resolved states observed in the single QPC.

In spin-polarized transverse magnetic focusing, the electron spin polarization is measured as additional voltage modulation across the collector QPC^26^ described by

2 $${V}_{c}=\frac{\gamma {I}_{e}}{{G}_{0}}(1-{P}_{e}{P}_{c})$$

where *γ* denotes the spin-independent efficiency parameter for collecting the emitted current, *I*_e_ expresses the emitter current, *G*_0_ is (2*e*^2^*/h*), ${P}_{e}=({I}_{e\uparrow }-{I}_{e\downarrow })/({I}_{e\uparrow }+{I}_{e\downarrow })$ stands for the spin polarization in the emitter QPC, ${P}_{c}=({I}_{c\uparrow }-{I}_{c\downarrow })/({I}_{c\uparrow }+{I}_{c\downarrow })$ represents the spin selectivity in the collector QPC, and *I*_eσ_ and *I*_cσ_ (*σ* = ↑ or ↓) respectively represent the emitter and collector currents with *σ* spin polarization. The initial spin polarization in the emitter QPC is defined as *P*_e_ > 0. We assumed that the conductance of both the emitter and collector was less than 1.0(2*e*^2^*/h*). Details of the theoretical description for *V*_c_ are given in Supplementary Information. Spin detection using this technique was first conducted in GaAs-based lateral QPCs without considering spin precession or spin-momentum locking^26^. In present InGaAs-based QPCs, because spin orientation transmitting the QPC depends on the electron momentum direction, a lateral QPC with emitter and collector of opposite momentum is best-suited to detecting spin manipulation by spin-momentum locking. When the focused spin orientation becomes opposite to the initial spin polarization (*P*_e_ = −*P*_c_), *V*_c_ increases because of the higher probability of spin transmission across the collector QPC. However, either the unpolarized current (*P*_e_ = *P*_c_ = 0) or the unresolved spin in one QPC (*P*_e(c)_ ≠ *P*_c(e)_ = 0) is responsible for the missing second term in equation (2), which results in the non-enhancement of *V*_c_. This lack of enhancement makes it possible to detect the relative spin orientation between the initial and final spin states by comparing the modulation amplitude of *V*_c_ between unpolarized and spin-polarized currents.

**Figure 5:**
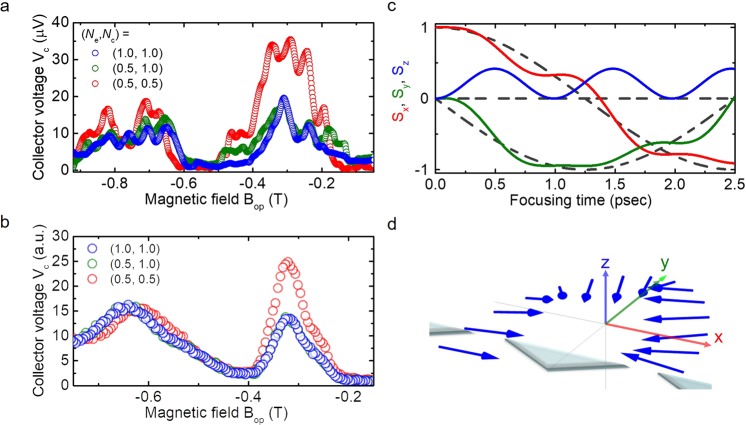
Experiment of spin-polarized transverse magnetic focusing and Monte Carlo simulation. (**a**) Collector voltage *V*_c_ as a function of out-of-plane magnetic field *B*_op_ for different quantized channel conditions. (*N*_e_, *N*_c_) denotes quantized conductance in units of 2*e*^2^*/h* as (0.5, 0.5) as red, (0.5, 1.0) as green, and (1.0, 1.0) as blue circles. (**b**) Monte Carlo simulation for the spin-polarized magnetic focusing for (i) both QPCs are spin-resolved, with (*N*_e_, *N*_c_) = (0.5, 0.5), (ii) emitter QPC is only spin polarized, with (*N*_e_, *N*_c_) = (0.5, 1.0), and (iii) both QPCs are not spin-resolved, with (*N*_e_, *N*_c_) = (1.0, 1.0). (**c**) Time evolution of spin vector components (*S*_x_(*t*), *S*_y_(*t*), *S*_z_(*t*)) at the first focusing peak. Spin components *S*_x_, *S*_y_, and *S*_z_ are calculated during the focusing time (2.5 ps) from emitter QPC to collector QPC. Dotted lines correspond to the trajectory of the adiabatic limit where the spin precession frequency is much faster than the cyclotron frequency. (**d**) Spatial evolution of spin orientation calculated in (**c**) along the semicircular orbital trajectory.

We performed spin-polarized magnetic focusing by setting quantized conductance to various values in the emitter and the collector QPCs, such as *N*_e_ × (2*e*^2^*/h*) and *N*_c_ × (2*e*^2^*/h*), respectively. Figure 5a shows the *B*_op_ dependence of *V*_c_ for (*N*_e_, *N*_c_) = (0.5, 0.5), (0.5, 1.0), and (1.0, 1.0) under *V*_TG_ = 4.0 V at *T* = 1.5 K (*N*_s_ = 2.0 × 10^12^ cm^−2^ and *α* = 5.96 × 10^−12^ eVm). The maximum voltage we applied to the top gate *V*_TG_ is 4.0 V to prevent the leakage current of the gate insulator. The resolution of perpendicular magnetic field *B*_op_ is about 1.3 mT. Two *V*_c_ peaks are readily apparent for the unpolarized single channel, *i.e*. (*N*_e_, *N*_c_) = (1.0, 1.0), at around −0.3 and −0.7 T, both with similar amplitudes (blue circles in Fig. 5a). The overlapping of multiple small peaks with the main signal is mainly attributable to the quantum interference. We do not observe or resolve the peak splitting on the signal around −0.3 T by the splitting of Rashba SO band^17^, which is presumably due to the effect of quantum interference and limited *B*_op_ resolution. The next step is to make both QPCs spin resolved, *i.e*. (*N*_e_, *N*_c_) = (0.5, 0.5), as shown by the red circles in Fig. 5a. The first peak voltage at *B*_op_ = −0.3 T is enhanced significantly. According to equation (2), this enhanced *V*_c_ originates from the second term, which is evidence that the finite spin polarization *P*_c_ in focused electrons has taken the opposite sign from that of the emitted spin polarization *P*_e_. To validate equation (2) further, we set the conductance to create a spin polarized emitter QPC and an unpolarized collector QPC, *i.e*., (*N*_e_, *N*_c_) = (0.5, 1.0) (green circles in Fig. 5a). In this case, neither of the *V*_c_ peaks is enhanced. The similarity of the amplitude for *V*_c_ to that with unpolarized current indicates the absence of the second term in equation (2). The peak position in the focusing signal shows rather robust under the asymmetric *V*_SR_ and *V*_SL_ conditions, because the maximum peak shift between (*N*_e_, *N*_c_) = (1.0, 1.0) and (0.5, 1.0) is expected to be 6.7 mT^34^ (Details are in Supplementary Information). By assuming *P*_c_ = −*P*_e_ at (*N*_e_, *N*_c_) = (0.5, 0.5), the estimated spin polarization becomes |*P*_c_| = |*P*_e_| = 0.81 ± 0.08, which is consistent with spin polarization in the single QPC device^27^. The short detection time of 2.4 ps from emitter to collector QPCs suggests that spin relaxation is not likely to occur during focusing^35^. Because electron spin experiences a large Rashba SO field *B*_so_ = 17.6 T, the time scale of spin precession because of the SO field is ${\tau }_{so}=\frac{2\pi \hslash }{g{\mu }_{B}{B}_{eff}}$ = 0.97 ps with *g* = 4.2^27^, which is shorter than the cyclotron travelling time of ${\tau }_{c}=2\pi \frac{{m}^{\ast }}{e{B}_{c}}$ = 4.8 ps with m^*^ = 0.04^27^. This result meets the requirements for adiabatic spin rotation, *i.e*. the regime of spin-momentum locking. The observed high spin polarization in both QPCs, together with the adiabatic spin rotation, suggests that spin orientation flips completely during the orbital trajectory. This result strongly suggests that spin manipulation by spin-momentum locking takes place during magnetic focusing.

### Comparing the focusing signal with results of Monte Carlo simulation

For further confirmation of spin manipulation by spin-momentum locking, we used the Monte Carlo (MC) method to simulate both the collector signals and spin orientation during focusing^36^. We calculated the ballistic spin transport based on the billiard ball model under the SO field (MC simulation details are presented in Supplementary Information). Figure 5b shows the calculated *V*_c_ signals in these experimental conditions: (i) both QPCs were spin-resolved, with (*N*_e_, *N*_c_) = (0.5, 0.5), (ii) only the emitter QPC was spin-resolved, with (*N*_e_, *N*_c_) = (0.5, 1.0), and (iii) both QPCs were unpolarized, with (*N*_e_, *N*_c_) = (1.0, 1.0). The emitter spin polarization was set at *P*_e_ = +1. Although the amplitudes of the *V*_c_ signals in the first and second peaks for (ii) and (iii) were similar, corresponding to (*N*_e_, *N*_c_) = (0.5, 1.0) and (1.0, 1.0), only the first focusing peak was enhanced when both QPCs were spin-resolved. The *V*_c_ signals calculated using the model show good agreement with experimentally measured signals. From the enhanced first peak, we calculated the collector spin polarization as |*P*_c_| = 0.87, which also shows good agreement with the experimental value. To elucidate the spin dynamics on the focusing trajectory, we calculated the time evolution of the spin vector (*S*_x_(*t*), *S*_y_(*t*), *S*_z_(*t*)) at the first peak (Fig. 5c). Initial spin polarization proceeded along the +*x*-axis (Fig. 5d). The time evolution of the spin vector was calculated at *B*_op_ = −0.315 T until the electrons reached the collector (*t* = 2.5 ps). As the calculated spin vector in Fig. 5d shows, the spin orientation remained perpendicular to the momentum direction with small periodic oscillations. Comparison with the trajectory of the spin-momentum locking regime, *i.e*. the adiabatic limit (dotted lines in Fig. 5c), demonstrates that a strong SO field determines the spin trajectory, forcing it to follow the adiabatic limit. It eventually results in the flipping of the spin orientation. Both the experiments and the MC calculations indicate clearly and unambiguously that the enhanced *V*_c_ results from spin manipulation by spin-momentum locking.

## Discussion

Spin manipulation by spin-momentum locking in the present experiment and simulation demonstrates the continuous change of the spin orientation during ballistic electron transport. The ballistic transport is the key to control the electron orbital trajectory and to realize a large Rashba SO field thanks to the Fermi velocity, which is distinguished from the Edelstein effect^37^ and inverse spin Galvanic effect (SGE)^38^ where biased electric field is responsible for the spin generation and detection. In the case of the Edelstein effect and inverse SGE^13^, by application of the bias electric field *E*, spin-up and/or spin-down Fermi circles are shifted as *δp* = m^*^μ_e_ *E*, where m^*^ signifies the electron effective mass and *μ*_e_ stands for the electron mobility. This *E* field induces the imbalance of spin-up and spin-down populations, resulting in spin generation under drift electron motion. The orientation of induced spin polarization is determined by the spin-momentum locking. In other words, spins are oriented perpendicularly to the applied *E* field direction. By contrast, spin manipulation by spin-momentum locking reveals how the spin rotates along the semi-circular trajectory as the electron momentum rotates (Fig. 1c). Actually, the shift of the Fermi circle is not necessary for spin manipulation by spin-momentum locking because the electron moves ballistically along the semi-circular orbital path. Therefore, the findings of this study highlight the importance of the electron trajectory for controlling the spin phase through spin-momentum locking.

To manipulate and detect spin orientation by spin-momentum locking, two functionalities are necessary for spin polarization and detection in the QPC. The first is to detect the opposite spin orientation from the initial spin in the collector QPC (Fig. 1a). The second is the initial spin orientation to be parallel to the SO field direction (blue arrow in Fig. 1c). In the case of conventional QPC structures studied previously^30,31,33,39–43^, the spin orientation does not change with electron momentum because of breakage of the time reversal symmetry, *i.e*., application of external magnetic fields^30,31,33,39–41^. For a QPC based on lateral SO interaction^44^, spin orientation becomes perpendicular to the QW plane. In such a case, spin precessional motion is rather induced under Rashba SO interaction. Recent finding by using both Rashba and lateral SO interactions focused on the coherent spin precession around Rashba SO field for spin-up and spin-down electrons separated by the magnetic focusing^34^. In order to induce the spin precessional motion, initial spin orientation is tilted from the direction of Rashba SO field by introducing the lateral SO interaction. In the present QPC structure, in-plane and collinear spin polarization to the Rashba SO field was generated through the emitter QPC to follow the spin orientation along Rashba SO field. For spin detection, spin selectivity in the QPC depends on the electron momentum direction because the spin splitting originates from the momentum-dependent SO field. Therefore, the opposite spin orientation was detected from the initial spin by changing the electron momentum direction. We use these advantages in the present QPC to demonstrate spin manipulation by spin-momentum locking.

**Figure 6:**
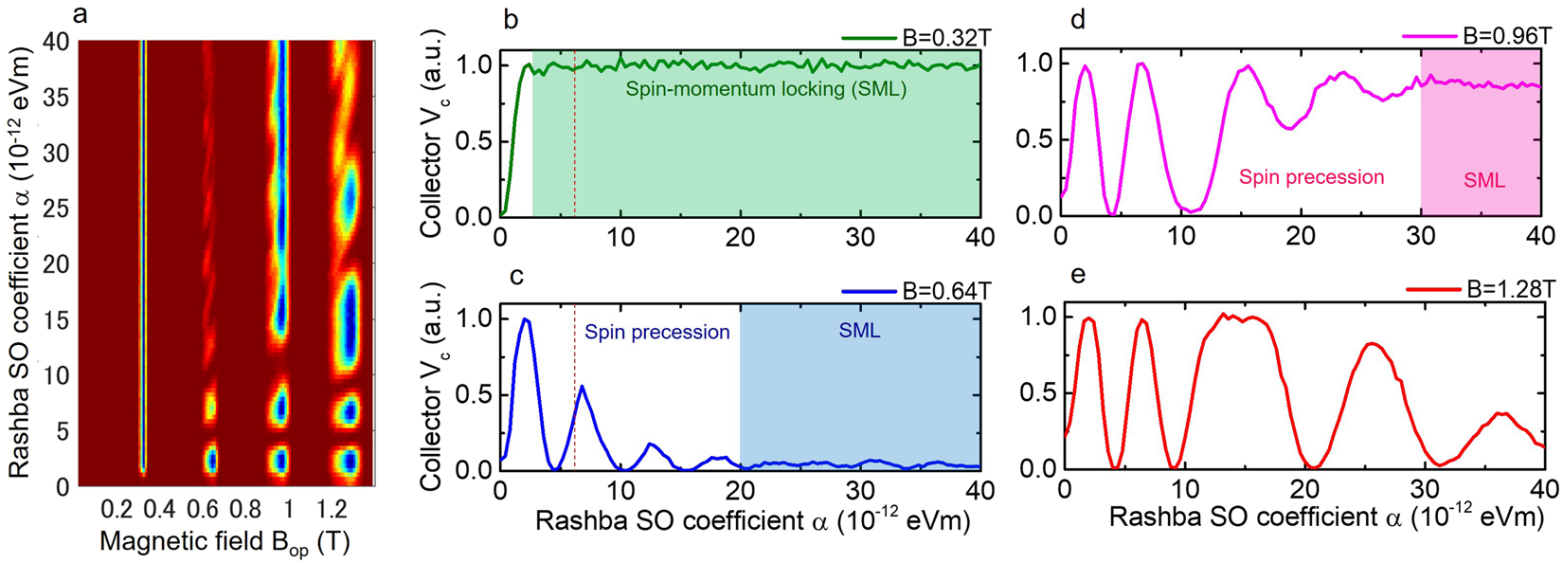
Simulated focusing signals as functions of magnetic field *B*_op_ and Rashba SO coefficient *α*. (**a**) 2D colormap of the focusing signal simulated by Monte Carlo simulation. Simulated voltage signal *V*_c_ at focusing peak *B*_op_ = (**b**) 0.32, (**c**) 0.64, (**d**) 0.96 and (**e**) 1.28 T as a function of Rashba SO coefficient *α*. Color-shaded region corresponds to the condition of spin-momentum locking as noted by SML.

Based on the argument of spin manipulation by spin-momentum locking together with momentum-dependent spin selectivity in the present QPC, we expect the suppressed signal in the second focusing around *B*_op_ = −0.7 T, because the refocused electron trajectory after scattering at the sample edge further rotates the spin orientation by 180°. In Fig. 5a,b, however, the relatively large signal in the second focusing is observed both in the experiment and the MC simulation. In order to gain more insight on the observed focusing peak, we plot 2D colormap of the simulated focusing voltage as functions of extended magnetic field and Rashba SO coefficient in Fig. 6a (Parameters for MC simulation are presented in Supplementary Information). Four focusing signals are observed around *B*_op_ = 0.32, 0.64, 0.96 and 1.28 T, where the voltage peak corresponds to the dark blue color in the 2D map. In the focusing signal at *B*_op_ = 0.32 T, no peak signal is initially observed in Rashba SO coefficient *α* < 2 ⋅ 10^−12^ eVm, then the signal changes to be constant peak line from *α* = 2 to 40 ⋅ 10^−12^ eVm. However, the signals at *B*_op_ = 0.64, 0.96 and 1.28 T initially show periodic oscillations as Rashba SO coefficient increased and saturate to the constant values in large *α*. We further plot the simulated voltage signal *V*_c_ at *B*_op_ = 0.32, 0.64, 0.96 and 1.28 T as a function of Rashba SO coefficient *α* in Fig. 6b–e, respectively. In the figure, maximum *V*_c_ signal (*V*_c_ = 1.0) corresponds to the opposite spin orientation from the initial spin in the collector QPC, while minimum *V*_c_ signal (*V*_c_ = 0) means the parallel spin orientation. In Fig. 6b, the *V*_c_ at *B*_op_ = 0.32 T, which corresponds to the first focusing signal, remains constant around *V*_c_ = 1.0 when *α* > 2 ⋅ 10^−12^ eVm, indicating the condition of spin-momentum locking. In Fig. 6c, the *V*_c_ at *B*_op_ = 0.64 T, *i.e*. the second focusing signal, oscillates until around *α* = 20 ⋅ 10^−12^ eVm, then the *V*_c_ is suppressed to be around zero. The suppression of *V*_c_ in large *α* values originates from the parallel spin orientation for emitted and collected electron spins, which also satisfies the condition of spin-momentum locking. It is worth noting that the condition of spin-momentum locking in the second focusing signal is shifted to larger Rashba SO coefficient than that of the first focusing signal. In the present magnetic focusing experiment, Rashba SO coefficient is around *α* = 6.0 ⋅ 10^−12^ eVm (the red dotted line in Fig. 6b,c), where the second focusing signal is not in the condition of spin-momentum locking. The relatively large signal in the second focusing originates from the fact that the *V*_c_ is oscillating due to the spin precessional motion. This indicates that we could analyze both spin manipulation by spin-momentum locking and spin precessional motion by Rashba SO field in the single device.

Spin manipulation by using SO field has remained limited to inducement of spin precession^45–48^ and coherent spin rotation such as electron spin resonance^49–52^. The spin manipulation through electron orbital motion presented here does not induce spin precessional motion around the magnetic field, minimizing the spin dephasing. Thereby, present demonstration indicates the potential scope of spin-momentum locking to provide more flexibility in the design of spintronic devices, fundamental spin functionality to future topological electronics and processing of quantum information through a geometrical phase^53–55^.

## Methods

### Device Structure and Fabrication

An InGaAs-based two-dimensional electron gas was grown epitaxially using metal–organic chemical vapour deposition. The layer structure from the bottom was (001) semi-insulating InP substrate, with 200-nm In_0.52_Al_0.48_As as a buffer layer, 6-nm In_0.52_Al_0.48_As as a carrier supply (Si doping with *N*_d_ = 4 × 10^18^ cm^−3^), 15-nm In_0.52_Al_0.48_As as a spacer layer, 5-nm (In_0.53_Ga_0.47_As)_0.41_(InP)_0.59_, 10-nm In_0.8_Ga_0.2_As as a QW, and 3-nm (In_0.52_Al_0.48_As)_0.3_(In_0.53_Ga_0.47_As)_0.7_/25-nm In_0.52_Al_0.48_As. Quaternary alloy layers for both sides of In_0.8_Ga_0.2_As QW enhance the interfacial Rashba SO contribution, which becomes necessary to manage the large Rashba SO field and long mean free path to satisfy the adiabatic limit of spin rotation. An epitaxial wafer was processed into trench-type single and lateral QPCs using electron beam lithography and reactive ion etching techniques. The QPC constriction shape was designed to induce a spatial gradient in the SO field, enabling electronic spin polarization and detection using the Stern–Gerlach spin filter. Atomic layer deposition of 150 nm Al_2_O_3_ was used to cover the surface and fill trenches for enhanced gate controllability. For the top-gate electrode, 5-nm Cr/150-nm Au was evaporated and lifted off. For ohmic contact, 200-nm AuGeNi was evaporated and annealed at 275 °C for 7 min. Carrier density, mobility and the SO coefficient were evaluated using a Hall bar connected to QPCs in series. The mean free path *l*_e_, measured as 0.16–3.14 μm, was dependent on the top-gate bias voltage *V*_TG_ (from −4.0 to +4.0 V). The SO coefficient evaluated from the beating pattern of the SdH oscillation and weak antilocalization shows *α* = 5.9–11.6 × 10^−12^ eVm, which corresponds to the SO field *B*_so_ from 12.5 to 18.5 T. Additionally, we measured the basic properties of the two-dimensional electron gas independently in a gate-fitted Hall bar with 20 μm width and 80 μm length. The widths and lengths of the channel in single and lateral QPCs were, respectively, 200–400 nm and 500 nm. The distance between the emitter and the collector in the lateral QPC was designed to be *d*_EC_ = 1.4 μm to satisfy the ballistic electron motion during focusing. Quantized conductance was measured by sweeping the side-gate bias voltage under the constant *V*_TG_. We additionally applied a dc source–drain bias voltage *V*_sd_ in the subband spectroscopy measurement. For transverse magnetic focusing, voltage *V*_c_ across collector QPC was measured under a fixed emitter current *I*_*e*_ = 100 nA. The external magnetic field *B*_op_ was applied perpendicularly to the QW plane between −1 T < *B*_op_ < +2.5 T for the focusing experiment. The measurement temperature was *T* = 0.22–1.5 K.

## Supplementary information

Supplementary Information
