# Supplementary material for: Spin-momentum locked spin manipulation in a two-dimensional Rashba system
Source: Sci Rep. 2019 Feb 13;9:1909. doi: 10.1038/s41598-018-37967-9 (PMC6374388; doi:10.1038/s41598-018-37967-9)
Supplement: Supplementary file 1 — Supplementary Information [file 41598_2018_37967_MOESM1_ESM.pdf]

Supplementary Information

Spin-momentum locked spin manipulation in a two-dimensional Rashba system

Makoto Kohda[1,2,3], Takanori Okayasu[1], and Junsaku Nitta[1,2,3]

[1] Department of Materials Science, Tohoku University, 6-6-02 Aramaki-Aza Aoba, Aoba-ku, Sendai 980-8579, Japan
[2] Center for Spintronics Research Network, Tohoku University, 2-1-1 Katahira, Aoba-ku Sendai, 980-8577, Japan
[3] Center for Science and Innovation in Spintronics (Core Research Cluster), Tohoku University, 2-1-1 Katahira, Aoba-ku Sendai, 980-8577, Japan

I. Effect of the in-plane external magnetic field to the quantized plateau

In Fig. 3b in the main text, $0.5(2e^2/h)$ plateau is disappeared at B_{op} = 2 T. We consider that the disappearance of $0.5(2e^2/h)$ by perpendicular B_{op} field originates from the cyclotron motion of electrons. Since the B_{op} field is perpendicular to the quantum well (QW) plane, Lorentz force modulates electron orbital motion, mixing the spin-up and spin-down electrons. This results in the unpolarized current and disappearance of $0.5(2e^2/h)$ plateau in relatively low magnetic field. To test the validity of our consideration, we have conducted the additional magnetic field dependence on $0.5(2e^2/h)$ plateau by applying the external magnetic field B_{ex} in-plane to the QW and parallel to the Rashba spin-orbit (SO) field B_{so}. In this configuration, the external magnetic field modulates the spin splitting energy without changing the electron orbital motion. Figure S1 shows the quantized conductance with different in-plane B_{ex} fields between -8 T to +8 T at T = 3.8 K. The $0.5(2e^2/h)$ plateau is stably observed up to ±8 T, indicating that the spin gap energy is larger than the Zeeman energy at B_{ex} = 8 T (1.9 meV at 8 T) and consistent to the estimated spin gap energy (5-6 meV) in our previous research [S1].

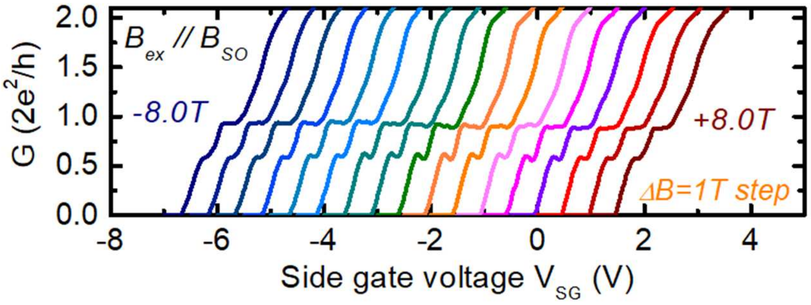

Figure S1. The quantized plateau with different in-plane magnetic field B_{ex} between -8 T and +8 T with 1 T step. Direction of B_{ex} is parallel to Rashba SO field B_{so}. Temperature is 3.8 K.

II. Theoretical description of collector voltage V_c for spin-polarized magnetic focusing under Stern-Gerlach type lateral QPCs:

In transverse magnetic focusing, electrons from the emitter quantum point contact (QPC) are ballistically focused to the collector QPC. Since the chemical potential in the collector QPC is increased due to electron accumulation, electrons start to flow back to the two-dimensional region, which induces voltage V_c, as described by,

$$V_c = \frac{I_c}{g_c} \quad \text{(S1)}$$

where I_c is the current injected into the collector and g_c is the conductance of the collector QPC. We assume that both the conductance in the emitter and collector QPCs are below $2e^2/h$. Then, by using spin-resolved transmission probability in the collector QPC, the collector voltage V_c is described by,

$$V_c = \frac{I_c}{\left(T_{c\uparrow} + T_{c\downarrow}\right)\left(\frac{e^2}{h}\right)} \quad \text{(S2)}$$

where $T_{c\sigma}$ is the transmission probability for σ (= ↑ or ↓) spin polarization with $0 \le T_{c\sigma} \le 1$. Spin polarization P_e in the emitter QPC and the spin selectivity P_c in the collector QPC are described by,

$$P_e = \frac{I_{e\uparrow} - I_{e\downarrow}}{I_{e\uparrow} + I_{e\downarrow}} \quad \text{(S3)}$$

$$P_c = \frac{I_{c\downarrow} - I_{c\uparrow}}{I_{c\uparrow} + I_{c\downarrow}} \quad \text{(S4)}$$

where $I_{e\sigma}$ and $I_{c\sigma}$ (σ = ↑ or ↓) are the emitter and collector currents, respectively, with σ spin polarization. Total emitter and collector currents are defined by $I_e = I_{e\uparrow} + I_{e\downarrow}$ and

$I_c = I_{c\uparrow} + I_{c\downarrow}$, respectively. It is noted that, due to the spin polarization/selectivity originating from the momentum dependent SO field, opposite spins are resolved for the emitter and collector QPCs. Details of the spin polarization mechanism are described in Ref. S1. Since the emitter current I_e is focused and transmitted through the collector QPC with spin resolution, I_c is described by,

$$I_c = I_{c\uparrow} + I_{c\downarrow} = \gamma\left(I_{e\uparrow}T_{c\uparrow} + I_{e\downarrow}T_{c\downarrow}\right) \quad \text{(S5)}$$

where γ is the spin independent transmission efficiency through the collector QPC due to imperfections in the focusing process by scattering and device geometry. Then, I_c is expressed using the spin polarization P_e of the emitter current as,

$$I_c = \gamma\left\{\left(\frac{1+P_e}{2}\right)I_e T_{c\uparrow} + \left(\frac{1-P_e}{2}\right)I_e T_{c\downarrow}\right\} = \frac{\gamma I_e}{2}\left[T_{c\uparrow} + T_{c\downarrow} + P_e\left(T_{c\uparrow} - T_{c\downarrow}\right)\right] \quad \text{(S6)}$$

As a result, the collector voltage V_c is expressed as,

$$V_c = \frac{\gamma I_e}{2\left(\frac{e^2}{h}\right)}\left[1 - P_e\left(\frac{T_{c\downarrow} - T_{c\uparrow}}{T_{c\uparrow} + T_{c\downarrow}}\right)\right] = \frac{\gamma I_e}{2\left(\frac{e^2}{h}\right)}\left(1 - P_e P_c\right) \quad \text{(S7)}$$

A theoretical description and an experiment have been reported for a GaAs/AlGaAs based lateral QPC [S2], where the spin polarization is induced by large in-plane B_{ip} field, but the momentum dependent spin polarization was not considered. In this experiment, parallel and collinear spin orientation between the emitter and collector QPCs were detected. However, Equation S7 indicates that antiparallel spin orientation between the emitter and collector QPCs enhances collector voltage while the parallel configuration decreases the amplitude of V_c.

III. Monte Carlo simulation of transverse magnetic focusing with strong Rashba spin-orbit (SO) interaction

When out-of-plane external magnetic field is applied to electrons moving in two-dimensional electron gas, electrons travel ballistically with a semicircular trajectory from the emitter to the collector due to the Lorentz force. The Rashba SO field is perpendicular to the electron momentum ***p***. We approximate the semicircular orbital to the polygonal trajectory with 100 apexes and calculate the electron propagation with momentum ***p*** for time intervals *dt* as well as the spin precession due to the Rashba SO field during this interval, and then recalculate the electron propagation with next ***p***. The magnitude of the electron momentum is fixed to satisfy energy conservation.

For simulating spin dynamics during transverse magnetic focusing, we assume electron spin behaves as a classical spin vector $\boldsymbol{S} = (S_x, S_y, S_z)$. The time evolution of the electron spin is described by $d\boldsymbol{S} / dt = \boldsymbol{\Omega} \times \boldsymbol{S}$, where $\boldsymbol{\Omega}$ includes the Rashba SO field and out-of-plane external magnetic field as,

$$\Omega = \begin{pmatrix} \dfrac{2\alpha k_F \sin\theta}{\hbar} \\ \dfrac{-2\alpha k_F \cos\theta}{\hbar} \\ \dfrac{g\mu_B B_{op}}{\hbar} \end{pmatrix} \quad \text{(S8)}$$

where α is the Rashba SO coefficient, k_F is the Fermi wave number, g is the Landé g factor, μ_B is the Bohr magneton, B_{op} is the out-of-plane magnetic field, $\hbar$ is the reduced Planck constant, and θ is the direction of the electron momentum defined from the $+x$ // [110] axis. The time evolution of the spin state does not affect orbital dynamics and vice versa. This semiclassical orbital dynamics corresponds to spin-independent orbital motion with an induced trajectory-dependent precession of spins [S3]. By solving the

differential equation of time evolution for a spin with initial spin polarization $\boldsymbol{S}_0 = (S_{x0}, S_{y0}, S_{z0})$ at t = 0 psec, the time evolution of each spin component is derived as,

$$S_x = \frac{1}{\omega^2}\left[A\left(AS_{x0} + BS_{y0} + CS_{z0}\right) + \left\{B^2 S_{x0} - ABS_{y0} + C\left(CS_{x0} - AS_{z0}\right)\right\}\cos(\omega t) - \omega\left(CS_{y0} - BS_{z0}\right)\sin(\omega t)\right] \quad \text{(S9)}$$

$$S_y = \frac{1}{\omega^2}\left[B\left(AS_{x0} + BS_{y0} + CS_{z0}\right) + \left\{-ABS_{x0} + A^2 S_{y0} + C\left(CS_{x0} - BS_{z0}\right)\right\}\cos(\omega t) - \omega\left(-CS_{y0} + AS_{z0}\right)\sin(\omega t)\right] \quad \text{(S10)}$$

$$S_z = \frac{1}{\omega^2}\left[C\left(AS_{x0} + BS_{y0} + CS_{z0}\right) + \left\{-C\left(AS_{x0} + BS_{y0}\right) + A^2 B^2 S_{z0}\right\}\cos(\omega t) - \omega\left(BS_{x0} - AS_{y0}\right)\sin(\omega t)\right] \quad \text{(S11)}$$

$$\omega = \sqrt{A^2 + B^2 + C^2} \quad \text{(S12)}$$

$$A = \frac{2\alpha k_F \sin\theta}{\hbar} \quad \text{(S13)}$$

$$B = \frac{2\alpha k_F \cos\theta}{\hbar} \quad \text{(S14)}$$

$$C = \frac{g\mu_B B_{op}}{\hbar} \quad \text{(S15)}$$

By considering the time evolution of the momentum direction due to the Lorentz force, we can calculate the spin vector after t psec with a semicircular orbital trajectory. 9×10^3 electrons are initially polarized along $+x$ // [110] axis, *i.e.* $\boldsymbol{S}_0$ = (1, 0, 0), and emitted from the QPC with an angle of electron momentum from 90º to 45º. Electrons experience isotropic impurity scattering under the trajectory and specular boundary scattering at the edge of the device. Non parabolicity in the band structure is not considered because of the small Fermi energy as a result of single subband occupation in the experimental condition. By the iterative process with dt time intervals, the electron trajectory as well as the spin orientation are calculated under the focusing process. We take into account the scattering probability during the electron trajectory as $N\times(dt/\tau_e)$, where τ_e is the momentum scattering time and $N\times dt$ corresponds to the propagating time from the emitter QPC (N is the increment number for the calculation).

When the electrons are focused to the collector QPC, electron spins are

transmitted through the collector QPC depending on the relative angle between **S** and *x* direction as described by,

$$\left|\left\langle \vec{x}_- \middle| \vec{S} \right\rangle\right|^2 = \sin^2\left(\frac{\theta}{2}\right) \quad \text{(S16)}$$

where the transmission probability becomes 1 when spin orientation is parallel to the – x direction. We calculated the probability of transmission as $\left|\left\langle \vec{x}_- \middle| \vec{S} \right\rangle\right|^2$ at the collector constriction where $\vec{x}_- = \frac{\sqrt{2}}{2}\begin{pmatrix} 1 \\ -1 \end{pmatrix}$ corresponds to the unit vector with S_x = -1 and $\vec{S}$ is the spin state after the semicircular trajectory. Then, we average $\left|\left\langle x - \middle| S \right\rangle\right|^2$ over all the electrons focused at the collector, and plot it as the collector signal by changing the out-of-plane external magnetic field B_{op}. Both the SO and external B_{op} fields were taken into account when simulating spin precession under the focusing. The parameters are determined according to the experimental result; the electron mean free path is l_e = 3 μm, the QPC width is 200 nm, the emitter and collector distance is d_{EC} = 1.4 μm, the carrier density is $N_s = 1.9\times10^{12}$ cm^{-2}, and the Rashba SO coefficient is $\alpha = 6.0\times10^{-12}$ eVm.

Before simulating our experimental results, we first calculated the magnetic focusing signal without SO interaction to ensure our billiard ball model reproduced the results of the GaAs/AlGaAs lateral QPC in an earlier experiment [S2]. We consider the magnetic focusing experiment in Ref. S2, where the spin polarization is induced by an in-plane external magnetic field and small out-of-plane external magnetic field focuses spin polarized electrons from emitter to collector QPCs. There is no spin precession taken into account due to the small SO field of a GaAs quantum well, and the device size considered in the calculation is according to our InGaAs-based magnetic focusing device. Then, we calculate the focusing signals of spin-polarized and unpolarized QPCs, which correspond

to (N_e, N_c), = (0.5, 0.5) and (1.0, 1.0) in units of ($2e^2/h$), respectively. As shown in Fig. S2, the focusing signal for (N_e, N_c), = (0.5, 0.5) is enhanced for both the first and second focusing peaks, whereas no enhancement was observed for (N_e, N_c), = (1.0, 1.0). This result is consistent over the experimental observations in Ref. S2 (Fig. 1(d)). The ratio of focusing peaks between spin-polarized and unpolarized signals in the MC simulation is 1.7~1.8, which is close to the predicted value of 2.0 in the theory. As a result, our billiard ball model for describing the magnetic focusing in good agreement with previous results. This ensures that the MC calculation captures the orbital trajectory by out-of-plane external magnetic field as well as the spin polarization/detection in the emitter and collector QPCs.

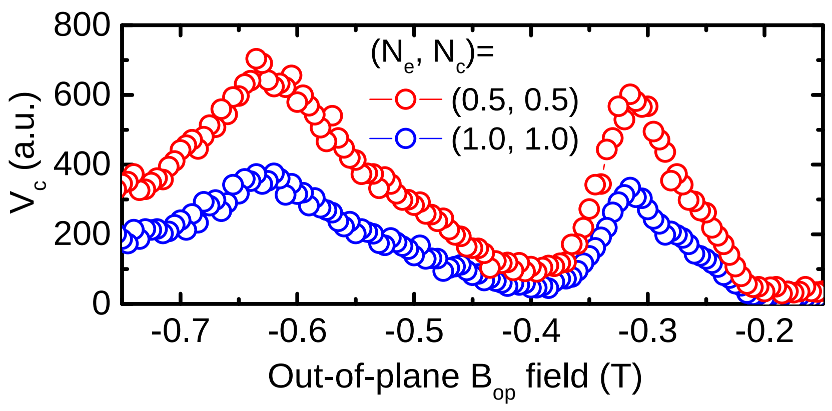

Figure S2. **Monte Carlo simulation of magnetic focusing in a GaAs/AlGaAs quantum well.** Calculated collector signal V_c as a function of out-of-plane magnetic field B_{op} for different quantized channel conditions of spin-polarized (red circles) and unpolarized (blue circles) QPCs, which correspond to (N_e, N_c), = (0.5, 0.5) and (1.0, 1.0) in units of ($2e^2/h$).

IV. Effect of the channel shift under the asymmetric bias condition

It is known that the asymmetric gate biasing would result in a lateral shift of the position of 1D channel, which changes the magnetic field at the focusing peak. we could expect the channel shift in the experimental data shown in Fig. 5a in the main text. Asymmetric gate bias voltage in our focusing device is defined as $\Delta V = V_{SR} - V_{SL}$, where V_{SR} and V_{SL} are right and left side-gate voltage in the QPC, and calculated to be 0.2, 1.7 and 1.0V for (N_e, N_c) = (1.0, 1.0), (0.5, 1.0) and (0.5, 0.5), respectively. Then, we approximate the position shift due to the asymmetric biasing according to Ref. S4

$$\Delta L = c\frac{W}{2}\frac{\Delta V}{|V_{SR}+V_{SL}|}, \quad \text{(S17)}$$

where W is the width of the split gate and c is the distribution of the electron density in the channel. By taking the value of c ~ 0.8 in Ref. S4, maximum ΔL is calculated to be 29 nm for largest asymmetric bias condition; W = 400 nm and $\Delta V = V_{SR} - V_{SL} = -3.8 - -5.5 = 1.7$ V. Since V_{SR} and V_{SL} are shared by two QPCs, we can also approximate $|V_{SR} + V_{SL}|/2$ and $\Delta V/2$ in Eq. S17. Based on the carrier density (2×10^{12} cm^{-2}) and Rashba SO parameter (6×10^{-12} eVm) in the present sample, the peak shift is approximately 6.7mT for the first focusing signals between (N_e, N_c) = (1.0, 1.0) and (0.5, 1.0). Such a small peak shift might be difficult to resolve in the present experiment since the first focusing signals for (N_e, N_c) = (1.0, 1.0) and (0.5, 1.0) in Fig. 5a in the main text show relatively broad peak structure whose full width at half maximum is approximately 120 – 150 mT.

V. Simulated condition for Monte Carlo simulation on second focusing signal

In Figs. 5a and 5b, we observed the relatively large signal on the second focusing both in the experiment and the MC simulation. Under the condition of spin-momentum locking, however, we expect the vanishingly small second focusing peak because of the anti-parallel spin orientation between the propagated spin and spin selectivity in the collector QPC [S5]. In order to make this ambiguous point clear, we conducted the MC simulation of the magnetic focusing in extended magnetic field range and Rashba SO coefficient shown in Figs. 6a – 6e in the main text. Simulated conditions are as follows: focusing radius 700 nm, QPC width 50 nm, Rashba coefficient $\alpha = 0$ - 40×10^{-12} eVm, perpendicular magnetic field 0 - 1.4 T, carrier density 1.9×10^{12} cm^{-2} and mean free path 3 μm. These parameters used in the simulation are similar values obtained in the experiment.

Supplementary references

[S1] Kohda, M. *et al.*, Spin-orbit induced electronic spin separation in semiconductor nanostructures. *Nat. Cmmun.* **3**, 1082 (2012).

[S2] Potok, R. M., Folk, J. A., Marcus, C. M. & Umansky, V. Detecting spin-polarized currents in ballistic nanostructures. *Phys. Rev. Lett.* **89**, 266602 (2002).

[S3] Zülicke, U., Bolte, J., & Winkler, R. Magnetic focusing of charge carriers from spin-split bands: semiclassics of a Zitterbewegung effect. *New. J. Phys*. **9**, 355 (2007).

[S4] Lo, S-T. *et al*. Controlled spatial separation of spins and coherent dynamics in spin-orbit-coupled nanostructures. *Nat. Commun*. **8**, 15997 (2017).

[S5] Usaj, G. & Balseiro, C. A. Transverse electron focusing in systems with spin-orbit coupling. *Phys. Rev.* B **70**, 041301(R) (2004).
